# Polyelectrolyte Complex Based Interfacial Drug Delivery System with Controlled Loading and Improved Release Performance for Bone Therapeutics

**DOI:** 10.3390/nano6030053

**Published:** 2016-03-22

**Authors:** David Vehlow, Romy Schmidt, Annett Gebert, Maximilian Siebert, Katrin Susanne Lips, Martin Müller

**Affiliations:** 1 Department of Polyelectrolytes and Dispersions, Leibniz Institute of Polymer Research Dresden, Hohe Strasse 6, Dresden D-01069, Germany; vehlow@ipfdd.de; 2 Department of Chemistry and Food Chemistry, Technical University Dresden, Mommsenstrasse 4, Dresden D-01062, Germany; 3 Department Chemistry of Functional Materials, Leibniz-Institute for Solid State and Materials Research Dresden, Helmholtzstrasse 20, Dresden D-01069, Germany; r.schmidt@ifw-dresden.de (R.S.); a.gebert@ifw-dresden.de (A.G.); 4 Institute of Experimental Trauma Surgery, Justus-Liebig-University, Schubertstrasse 81, Giessen 35392, Germany; maximilian.siebert@med.uni-giessen.de (M.S.); katrin.s.lips@chiru.med.uni-giessen.de (K.S.L.)

**Keywords:** polyelectrolyte complex, drug delivery, risedronate, rifampicine, attenuated total reflection Fourier transform infrared spectroscopy (ATR-FTIR), biocompatibility, hMSC

## Abstract

An improved interfacial drug delivery system (DDS) based on polyelectrolyte complex (PEC) coatings with controlled drug loading and improved release performance was elaborated. The cationic homopolypeptide poly(l-lysine) (PLL) was complexed with a mixture of two cellulose sulfates (CS) of low and high degree of substitution, so that the CS and PLL solution have around equal molar charged units. As drugs the antibiotic rifampicin (RIF) and the bisphosphonate risedronate (RIS) were integrated. As an important advantage over previous PEC systems this one can be centrifuged, the supernatant discarded, the dense pellet phase (coacervate) separated, and again redispersed in fresh water phase. This behavior has three benefits: (i) Access to the loading capacity of the drug, since the concentration of the free drug can be measured by spectroscopy; (ii) lower initial burst and higher residual amount of drug due to removal of unbound drug and (iii) complete adhesive stability due to the removal of polyelectrolytes (PEL) excess component. It was found that the pH value and ionic strength strongly affected drug content and release of RIS and RIF. At the clinically relevant implant material (Ti40Nb) similar PEC adhesive and drug release properties compared to the model substrate were found. Unloaded PEC coatings at Ti40Nb showed a similar number and morphology of above cultivated human mesenchymal stem cells (hMSC) compared to uncoated Ti40Nb and resulted in considerable production of bone mineral. RIS loaded PEC coatings showed similar effects after 24 h but resulted in reduced number and unhealthy appearance of hMSC after 48 h due to cell toxicity of RIS.

## 1. Introduction

The local functionalization of bone substitute materials (BSM) by adhesive drug delivery systems (DDS) is highly relevant for fracture healing and tissue regeneration within systemically altered bone (osteoporosis, multiple myeloma), since osteotherapeutic drugs like bisphosphonates and bone surgery relevant antibiotics have undesired side effects like osteonecrosis or bacterial resistance, respectively, when given systemically. Different polymer based nanoparticular DDS are known like pure [1,2] and hybrid liposomes [3,4], polymeric micelles [5,6,7,8] or hollow polymer capsules [9,10,11,12], which have beneficial properties in the volume phase with respect to defined loading, releasing and cell targeting. However these nanoparticular polymer based DDS were not primarily used as adhesive coatings to our knowledge.

Herein, as adhesive, DDS polyelectrolyte complex (PEC) particles were chosen, which can be used to modify model substrates and BSM locally [13,14]. General benefits of PEC are easy preparation in aqueous media under mild conditions, the absence of toxic solvents and the possibility to use biorelated compounds like polysaccharides and polypeptides. From those adhesive PEC coatings two model drugs, rifampicin (RIF) and risedronate (RIS), are aimed to be released in a defined and delayed manner. Herein as BSM novel Ti40Nb alloys, which are promising materials for osteosynthetic plates due to their mechanical properties [15], were selected. Nanoparticles based on PEC are known since longer times [16], where mixing polycation (PC) and polyanion (PA) solutions results in phase separation and the formed turbid dispersion contains nanoscaled colloid particles. The driving force of this process is claimed to be the release of the counterions of PC and PA rather than the electrostatic attraction [16,17]. At first small primary PEC particles are formed, which rapidly aggregate to the final stable secondary particles in the nano- up to micro-range [18]. An important experimental parameter is the ratio related to the molar concentration of anionic to cationic monomer units n^−^/n^+^. For n^−^/n^+^ > 1 the net charge of PEC particles is negative and for n^−^/n^+^ < 1 it is positive. Importantly, drugs can be integrated into PEC particles during or after the mixing process and they may be physically entrapped or bound by a variety of molecular interaction forces.

For clinical application the drug loaded PEC particles have to meet special requirements like colloidal stability, adhesive stability, controlled drug loading and release and biocompatibility. In previous studies we have addressed several of these requirements [13,14]. However, we could not determine the loading capacity of our drug/PEC systems and the drug release should be further delayed. Therefore, in this study we outline an improved preparation protocol based on centrifugation and redispersing of PEC dispersions to address also these requirements. Furthermore, the modification of Ti40Nb alloys, which is a mechanically favorable material for osteosynthetic plates, with drug loaded PEC coatings will be introduced and first results on drug release and biocompatibility given.

## 2. Experimental Section

### 2.1. Materials

**Figure 1:**
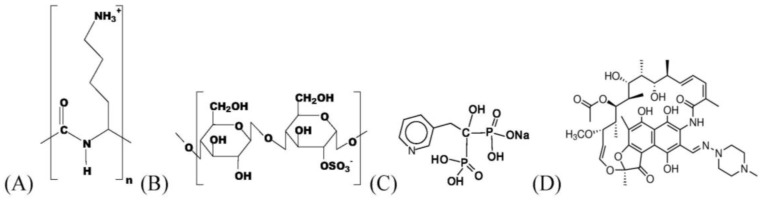
Scheme of the mainly used chemical compounds: Poly(l-lysine) (PLL) (**A**); Cellulose sulfates (CS-0.5) (**B**); Risedronate (RIS) (**C**); Rifampicin (RIF) (**D**).

Important chemical compounds are given in Figure 1. As polycation (PC) the linear poly(l-lysine) (PLL, 30,000–70,000 g/mol, Sigma Aldrich, St. Louis, MO, USA) with a degree of substitution (DS) = 1.0 was used. The cellulose sulfate with DS = 3.0 (CS-3.0, 1,200,000 g/mol, Janssen Chimica, Belgium) and cellulose sulfate with DS = 0.5 (CS-0.5, no molecular weight provided, Euroferm, Erlangen, Germany) are our polyanion (PA) components. These polyelectrolytes (PEL) were dissolved to 0.002 M solutions related to their monomer units. For equal molar charge units of PA and PC by complexation we use a mixture of high and low substituted CS which, corresponds to an average DS = 1.0 further denoted as CS-1.0. As drugs the antibiotic rifampicin (RIF, Carl Roth, Karlsruhe, Germany) and the bisphosphonate risedronate (RIS, Sigma Aldrich, St. Louis, MO, USA) were used. Buffer substances, tris(hydroxymethyl)-aminomethane (TRIS, Sigma Aldrich, St. Louis, MO, USA), 2-(4-(2-Hydroxyethyl)-1-piperazinyl)-ethane sulfonic acid (HEPES, Sigma Aldrich, St. Louis, MO, USA) and citric acid monohydrate (CIT, Sigma Aldrich, St. Louis, MO, USA), were dissolved to 0.01 M solutions and used as release media. Sodium chloride in various concentrations was also used as release media.

### 2.2. Preparation of Polyelectrolyte Complex (PEC) Particles

Binary (drug free) PEC particles were prepared by mixing defined volumes of 0.002 M PLL and CS-1.0 solutions resulting in defined molar mixing ratios n^−^/n^+^ = 0.5 to 1.5. The net charge of PEC dispersions was calculated by Equation (1) defining the stoichiometric mixing ratio n^−^/n^+^ related to the molar amount of anionic (n^−^) and cationic (n^+^) repeating units.

(1) $$n^{-}/n^{+}=\frac{\left(F_{\text{PA}}^{-}\cdot n_{\text{PA}}^{-}\right)}{\left(F_{\text{PC}}^{+}\cdot n_{\text{PC}}^{+}\right)}$$

$n^{-}/n^{+}$: molar mixing ratio
$F^{+/-}$: molar charge units of PA and PC
$n^{+/-}$: molar amount of anionic (n^−^) and cationic (n^+^) repeating units

Ternary drug loaded PEC were prepared by mixing defined volumes of PLL/drug (0.002 M/0.00025 M) and CS/drug (0.002 M/0.00025 M) mixtures resulting in defined molar mixing ratios n^−^/n^+^ = 0.3 to 1.7. For preparation of the binary PLL/RIS and CS/RIS mixtures RIF and RIS powders were given into PLL and CS solutions, respectively, and the pH was adjusted and kept constant at a defined pH value by the addition of HCl or NaOH (see above). No salt was given to the PLL, CS or drug solutions used for PEC preparation. For the calculation of PLL, CS and drug amounts used for the preparation of the drug loaded ternary PEC particles a modified equation was used, where additionally the charge amount of the anionic or cationic drug was considered. For negatively charged drugs Equation (2) and for positively charged drugs Equation (3) is valid.

(2) $$n^{-}/n^{+}=\frac{\left(F_{\text{PA}}^{-}\cdot n_{\text{PA}}^{-}\right)+\left(F_{\text{drug}}^{-}\cdot n_{\text{drug}}^{-}\right)}{\left(F_{\text{PC}}^{+}\cdot n_{\text{PC}}^{+}\right)}$$

(3) $$n^{-}/n^{+}=\frac{\left(F_{\text{PA}}^{-}\cdot n_{\text{PA}}^{-}\right)}{\left(F_{\text{PC}}^{+}\cdot n_{\text{PC}}^{+}\right)+\left(F_{\text{drug}}^{+}\cdot n_{\text{drug}}^{+}\right)}$$

### 2.3. Centrifugation of PEC Particles and Coating Procedure

**Figure 2:**
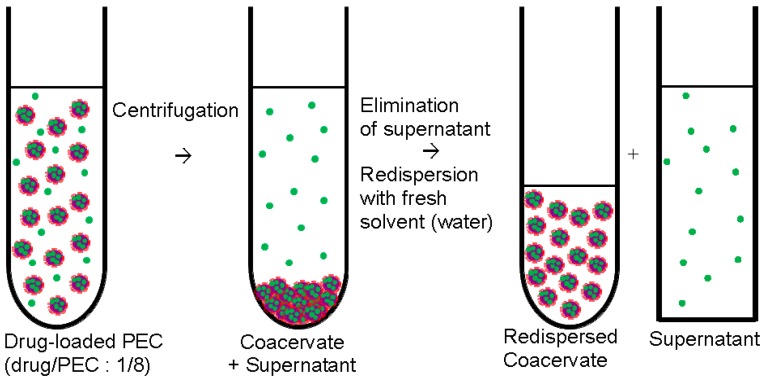
Centrifugation and redispersion of polyelectrolyte complex (PEC) particles (centrifugation procedure).

Drug/PEC dispersions were refined by centrifugation to separate the drug loaded PEC particles from excess components like the uncomplexed PEL or the unbound drug. The preparation protocol is given in the Figure 2.

After centrifugation with rotational speed of 10,000 rpm dense pellet phase (coacervate) at the bottom and the clear phase in the supernatant was obtained. Raw dispersion before centrifugation and the supernatant phase were further studied by ultraviolet/visible (UV/VIS) and CD spectroscopy (see below) to determine the loaded drug amount. Typically, 2 mL of raw dispersion was centrifuged and the coacervate phase was redispersed in 0.2 mL of Millipore water. The redispersed coacervate (0.2 mL) was solution casted onto the Ge model substrate (internal reflection element (IRE), see below) resulting in a homogeneous coating and dried at 50 °C in oven for 30 min. In these coatings the drug content was in the range of 100 μg. Given release media volumes of 4 mL (see below in Section 2.5) and assuming complete drug release, drug (RIS, RIF) concentrations of around 0.0001 M prevail, which are well below the saturation concentrations ensuring sink conditions.

### 2.4. Quantitative UV/VIS and CD Spectroscopy

UV/VIS and circular dichroism (CD) spectroscopy were used to determine the drug loading of PEC particles in the volume phase (i) and the drug release from PEC coatings (ii). Drug loading was determined by comparing the measured intensity of a given UV/VIS band of the drug (RIS or RIF) in the original solution with the respective intensity of the supernatant (depletion) of a drug loaded PEC NP dispersion after centrifugation and redispersion (see above). Drug release out of PEC coatings and the time dependence was determined by measuring the actual intensities of given UV/VIS bands of the drug in the release medium (enrichment) at defined times and calculating the respective concentrations based on drug solutions of known concentrations. Various release media (CIT, HEPES, TRIS buffer, respectively) were applied (see above). The measurements were performed at the spectropolarimeter J 810 (Jasco Labor- und Datentechnik GmbH, Groß-Umstadt, Germany) in 1 cm quartz glass cuvettes.

### 2.5. Quantitative ATR-FTIR Spectroscopy

Fourier transform infrared (FTIR) measurements were performed on a Vertex V70 spectrometer (Bruker Optics GmbH, Ettlingen, Germany) controlled by OPUS software (Bruker, Ettlingen, Germany) in the attenuated total reflection (ATR) mode using a single beam 4-mirror-ATR attachment (Perkin Elmer GmbH, Überlingen, Germany). FTIR spectra were recorded at a spectral resolution of 2 cm^−1^ and 100 scans per sample were coadded and averaged. Usually, at first reference intensity spectra *I*_R_ were recorded from the uncoated germanium internal reflection element (Ge IRE) and sample intensity spectra *I*_S_ were recorded from the Ge IRE coated by the redispersed PEC/Drug dispersion after drying (see above). Rationing *I*_S_ and *I*_R_ absorption spectra were obtained according to *A* = −log(*I*_S_/*I*_R_). The atmospheric water vapor compensation tool of the OPUS software was used resulting into well compensated absorbance spectra. The drug/PEC coated Ge IRE was placed in an *in situ*-cell, whose liquid chamber (volume: 4 mL) was filled by various release media (CIT, HEPES, TRIS buffer, respectively) and after defined times the release media were removed, the liquid chamber was rinsed twice with fresh water and dried in a gentle N_2_ stream. Thereafter, again I_S_ spectra were recorded and respective attenuated total reflection Fourier transform infrared spectroscopy (ATR-FTIR) absorption spectra computed. ATR-FTIR spectra were quantitatively analyzed with respect to PEC and drug content. The actual PEC content was quantified using factor analysis (see below), while the drug content was quantified by lineshape analysis (see below).

#### 2.5.1. Factor Analysis (FA)

Factor analysis of FTIR spectra is based on the linear combination of known factor spectra ($A_{\text{pure}\;\text{component}\;j}$) in order to represent the FTIR spectrum of a PEC sample with unknown composition ($A_{\text{PEC}\;\text{mixture}}$).

(4) $$A_{\text{PEC}\;\text{mixture}}=\overset{n}{\underset{j=1}{\sum }}f_{j}\cdot A_{\text{pure}\;\text{component}\;j}+X$$

$A_{\text{PEC}\;\text{mixture}}$: PEC absorbance spectrum
$f_{j}$: factor of the *j-th* pure component absorbance spectrum
$A_{\text{pure}\;\text{component}\;j}$: *j-th* pure component (factor) spectrum
*X*: residuals

**Figure 3:**
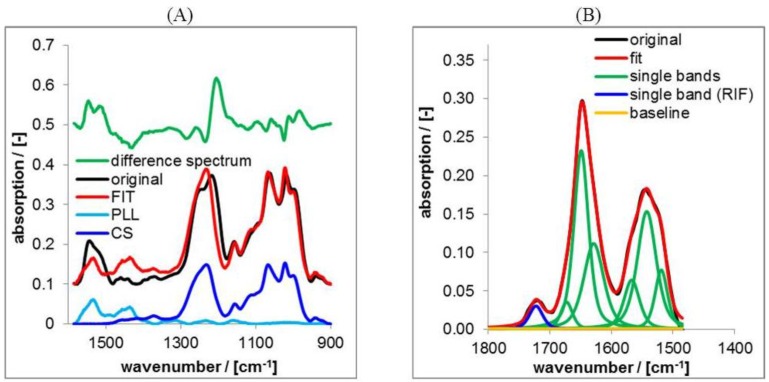
(**A**) Typical result of factor analysis on attenuated total reflection Fourier transform infrared spectroscopy (ATR-FTIR) spectra of casted films of PLL/CS-1.0; (**B**) Typical result of lineshape analysis (LSA) on ATR-FTIR spectra of casted films of PLL/CS-1.0 loaded by RIF.

In practice we used the command “Spectra Subtraction” in the OPUS 7.0 software, where the PLL and the CS spectrum was subtracted from respective PEC spectra of PLL/CS mixtures with the condition of minimum intensity of the difference spectrum (residuals). A typical result of this procedure applied to a spectrum of the PLL/CS-1.0 PEC system is shown in Figure 3A.

#### 2.5.2. Lineshape Analysis (LSA)

LSA is based on the superposition of band components (herein Lorentzian/Gaussian 50%/50%) to represent a measured lineshape as it was described for other systems therein [19]. LSA was performed on the ATR-FTIR spectrum of the PEC with drug and that of the PEC without drug. Typical results of LSA on RIF/PEC are presented in Figure 3B.

For the RIF/PEC system the drug content can be quantified directly by the isolated RIF band at around 1725 cm^−1^ as percentage value with respect to the initial RIF band intensity. The relative RIF content *Q*_RIF_ was calculated according to Equation (5).

(5) $$Q_{\text{RIF}}\;\left[\%\right]=\frac{A_{\text{RIF}}(t_{\text{actual}})}{A_{\text{RIF}}(t_{0})}\cdot 100\%$$

For the RIS/PEC system the analysis of the drug content is more difficult, since diagnostic band components of RIS (P=O stretching vibration) are overlapped by band components of CS (S=O stretching vibration). In this case, a methodology was applied in the lines of own former reports [20] which considers the background of diagnostic drug band components.

### 2.6. Colloid Titration

True molar charge units (F^+/−^) of PEL-solutions were measured by colloid titration using low molecular 0.001 M PDADMAC or PES-Na solutions as titrating solutions with F^+/−^ = 1.0. The particle charge detector (PCD-04 (Mütek), BTG, Eclepens, Switzerland) was used operated by software version 1.00.001 (BTG, Eclepens, Switzerland). The general analyzing protocol was published [21]. For analyzing the PEL/drug mixtures at extreme pH values (3, 10) we used the PCD in a manual mode to keep a constant pH value. In general F^+/−^ is expressed by the ratio between the true charged monomers and the monomer concentration. Usually, we averaged over three measurements for PEC preparation. In Table 1 the arithmetic average with its standard deviation of all measured molar charge units of the PEL are presented.

### 2.7. Dynamic Light Scattering (DLS)

Dynamic light scattering (DLS) was used to determine the particle sizes of PEC and drug/PEC particles. DLS measurements were performed at the Jianke Portable Particle Sizer (Jianke Instruments Co. Ltd., Wuhu, China) at a scattering angle of 90°. Cylindrical glass cuvettes with diameter of 10 mm were used, which were filled with 2 mL of PEC samples. The analysis of the measured autocorrelation functions was performed with the ALV-5000/E/EPP-Software of ALV GmbH, Langen, Germany to calculate DLS-parameters. The Stokes-Einstein equation was used to estimate the hydrodynamic radii or diameter. Furthermore, the intensity weighted particle size distribution was used, as it was applied earlier in [21]. The polydispersity index (PDI) was determined by fitting the semilog plot of the autocorrelation function *G*(*t*) by a power series according to log[*G*(*t*)] = a + b × *t* + c × *t*^2^]. The polydispersity index is defined as PDI = 2c/b^2^.

### 2.8. Scanning Force Microscopy (SFM)

Silicon nitride probe tips (Nanosensors, Darmstadt, Germany) having an apex of around 10 nm were used. Scanning force microscopy (SFM) images in topography, error and phase mode were recorded in non-contact-mode from the unloaded and drug loaded PEC NP coatings at silicon wafer substrates under ambient atmosphere using Nanostation II (BRUKER Nano GmbH, Karlsruhe, Germany). SFM scanning parameters were optimized by minimizing the amplitudes measured in the error mode. SFM images were postprocessed from SFM raw data using SISCANPro software (BRUKER Nano GmbH, Karlsruhe, Germany) or SPIP (software package for nano- and microscale image processing), Image Metrology, Horsholm, Denmark).

### 2.9. Cellular Compatibility

Human mesenchymal stem cells (hMSC) of four bone healthy donors were cultured from reaming debris that was collected during routine trauma surgery to stabilize fractures with osteosynthetic materials. The study was approved by the local ethic committee (74/09) and all patients give their written consent. They did not suffer from additional disease and were aged 22, 22, 25, and 63 years. Directly after operation the reaming debris was transferred into MesenProRS medium (Life Technologies, Carlsbad, CA, USA) in an incubator with 37 °C and 5% CO_2_. Cells were passaged 2–4 times and seeded with a density of 20,000 cm^2^ in 24-well plates that contained (a) pure cell culture plastic; (b) Ti40Nb alloy without modifications; (c) PEC at Ti40Nb; (d) pure RIS film at Ti40Nb; and (e) RIS-loaded PEC at Ti40Nb. After 24 h incubation time wells were controlled light microscopically (Type 090-135.002, Leica, Wetzlar, Germany) and documented by digital photography (Ds-Fi1, Nikon, Duesseldorf, Germany). Afterwards the medium was changed to osteogenic differentiation medium containing Dulbecco’s modified eagle medium (DMEM, low glucose, L-glutamine, PAN-Biotech, Aidenbach, Germany) with 10% fetal calf serum (FCS, Biochrom, Berlin, Germany), 10^−7^ M dexamethasone (Sigma-Aldrich, Munich, Germany), 10^−2^ M β-glycerol-phosphate hydrate (Sigma-Aldrich), 5 × 10^−5^ M sodium L ascorbate (Sigma Aldrich), 100 U/mL penicillin (Life Technologies), and 100 μg/g streptomycin (Life Technologies). After additional 24 h cells were documented again light microscopically.

## 3. Results and Discussion

Herein, results on the colloid, interfacial and drug carrier properties of unloaded polyelectrolyte complex (PEC) particles and those loaded by low molecular therapeutics for bone diseases are given and discussed in the following, aiming at further clinical applications. This study is focused on a PEC system composed of cationic poly(L-lysine) (PLL) combined with anionic cellulose sulfate (CS) of high and low sulfation degree, which is loaded by either the anionic bisphosphonate risedronate (RIS) or the zwitterionic antibiotic rifampicin (RIF). This study is an extension of previous work by us [13,20,22], where cationic poly(ethyleneimine) (PEI) and CS or anionic dextran sulfate were combined and loaded by other anionic drugs, namely the bisphosphonates pamidronate, zoledronate and simvastatin. While the accent was more focused on the protective integration of drugs into PEC coatings, the release kinetics of these previous systems was too fast. To address this problem, a new preparation protocol was elaborated now, which is based on centrifugation of the drug/PEC system. In the following we will present beneficial results with respect to the colloid, interfacial and release properties of RIS and RIF loaded PEC particles arising from this new preparation concept.

### 3.1. Drug Free PEC Systems

#### 3.1.1. Colloid Properties

**Figure 4:**
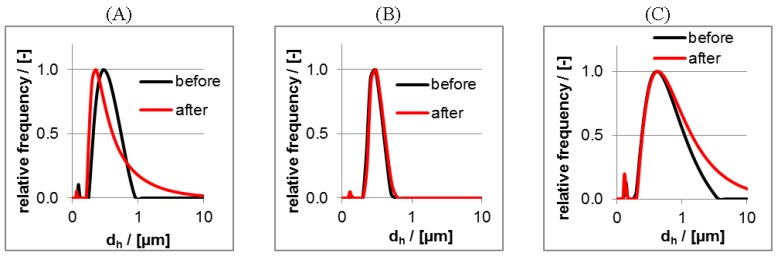
Intensity weighted size distributions from dynamic Light Scattering (DLS) measurements on dispersions of PLL/CS-3.0 (**A**); PLL/CS-1.0 (**B**) and PLL/CS-0.5 (**C**) (all PEC 0.9 samples).

In the Figure 4 size distributions of PEC particles without drugs based on DLS measurements are shown for PEC 0.9 of the PLL/CS systems for CS with three different charge factors F^−^ according to pure CS-0.5 and CS-3.0 and a mixture of both.

For the PLL/CS-1.0 system (Figure 4B) both very similar and rather narrow size distributions before and after centrifugation were observed in contrast to the PLL/CS-0.5 (Figure 4A) and PLL/CS-3.0 (Figure 4C) system, for which the size distributions broadened or shifted in its maximum after centrifugation. Equal size distributions before and after centrifugation might be interpreted as unaltered colloidally stable PEC particles being completely redispersible after precipitation by centrifugation. In the other two cases (Figure 4A,C) the PEC particles altered with respect to size or size distribution, which can be generally interpreted by less colloidal stability. Reasons might be effects on the conformation of the PEL or on the aggregation state of the PEC particles upon centrifugation, which might occur favorably, when the charge densities of the mixed PEL are very different like for PLL and CS-3.0 or CS-0.5. Obviously, it plays a role, whether polycation and polyanion solution have around the same concentration of charged repeating units like for PLL/CS-1.0 or whether they have different ones like for PLL/CS-0.5 and PLL/CS-3.0. Additionally, there might be a volume effect, since for PLL/CS-1.0 the polycation/polyanion volumes to be mixed are around equal but for PLL/CS-0.5 and PLL/CS-3.0 they are very different. Presumably, the mixing process using very different volumes and charged unit concentrations of polycation and polyanion solution might be more locally and kinetically controlled compared to that using equal volumes and charged unit concentrations.

Furthermore, the redispersibility of a PEC precipitate after centrifugation is dependent on the interplay of electrostatic and hydrophobic interaction between the PEL of the PEC system, which was shown for the PDADMAC/PMA-MS system composed of a synthetic strong polycation with rather hydrophilic and a synthetic weak polyanion with considerable hydrophobic character [21]. Presumably, the herein investigated PLL/CS-1.0 system provides a similar balance between charge density and hydrophobicity of its PEL components enabling the formation of small primary PEC particles (*R*_H_ = 5–10 nm) and the further aggregation of those to secondary PEC particles (*R*_H_ = 100–250 nm), as it was described therein [18,21]. Similar to those, the PLL/CS PEC particles of this study have sizes in the nano-range as it is shown in the Table 2.

At first, the PEC 1.1 (n^−^/n^+^ = 1.1, anionic) systems have larger hydrodynamic radii than the PEC 0.9 (n^−^/n^+^ = 0.9, cationic) systems of PLL/CS, respectively. Secondly, for the PLL/CS-3.0 and PLL/CS-0.5 systems centrifugation has a significantly larger effect with respect to countrate and hydrodynamic radii, while for the PLL/CS-1.0 system such effect was lowest. Because of this obvious colloidal stability upon centrifugation, the PLL/CS-1.0 system was selected for further experiments, where additionally drugs are integrated into PEC particles. Furthermore, for all six systems the polydispersity index increased upon centrifugation, which might be due to the typically high polydispersities known for uncomplexed biorelated polymers like the herein used PLL and CS.

#### 3.1.2. Adhesive Properties of PEC Coatings

**Figure 5:**
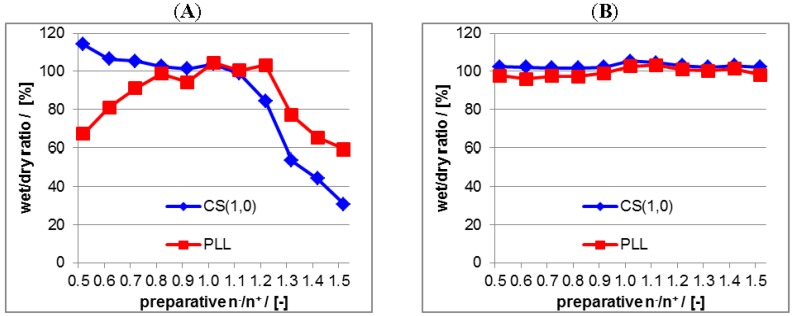
(**A**) Ratio between PEL amount before and after water-rinsing step in the dry state due to ATR-FTIR measurements of PEC coatings from non-centrifuged PEC dispersions at germanium internal reflection element (Ge IRE); (**B**) Ratio between PEL amount before and after water-rinsing step in the dry state due to ATR-FTIR measurements of PEC coatings from centrifuged PEC dispersions at Ge IRE.

As the second important property films of centrifuged PEC particles were checked for adhesive stability at model substrates. Such an adhesive stability of the PEC coating at bone substituting materials (BSM) is highly relevant for drug delivery in clinical applications, since only the drug and not compounds of the matrix should be released in the biological environment peripheral to the BSM. Herein, a germanium (Ge) substrate was chosen as model system, since it is commonly used as standard internal reflection element (IRE) in *in situ*-FTIR measurements. These Ge substrates were coated with PLL/CS complexes by casting PEC dispersions and ATR-FTIR spectra on these coatings in the initial dry state and the dry state after rinsing in water for 5 min were recorded. Comparing ATR-FTIR spectra measured before (“dry”) and after rinsing (“wet”) provided a measure for the adhesive stability of PLL/CS complexes and their individual PEL components (PLL, CS), which was introduced [20]. In detail PEL amounts before and after rinsing in the dry state were obtained by ATR-FTIR spectroscopy applying factor analysis (FA, see Experimental Section 2.5.1) defining a certain wet/dry ratio. In the Figure 5 this wet/dry ratio is plotted *versus* the preparative mixing ratio n^−^/n^+^ for PLL/CS complexes as a function of n^−^/n^+^.

Figure 5A is related to the uncentrifuged PLL/CS system and Figure 5B to the centrifuged PLL/CS system. For the uncentrifuged PLL/CS system both the bound PLL and CS amount does not keep stable within the whole range of n^−^/n^+^ = 0.5–1.5. The bound CS amount is only stable (wet/dry ratio ≈ 100%) from n^−^/n^+^ = 0.5–1.1 and the bound PLL amount only within n^−^/n^+^ = 0.8–1.2. At mixing ratios deviating much from n^−^/n^+^ = 1.0 (charge stoichiometry) the adhesive stability is decreasing (wet/dry ratio << 100%). For the centrifuged PLL/CS system both the bound PLL and CS amount keeps constant at 100% within the total n^−^/n^+^ range, which confirms complete adhesive stability of the PLL/CS coating. Hence, we conclude from these data that in the centrifuged PLL/CS system, regardless of the preparative mixing ratio n^−^/n^+^, the excess component (PLL for PEC with n^−^/n^+^ < 1 and CS for PEC with n^−^/n^+^ > 1 is removed by centrifugation and the casted PEC coating contains predominantly PEC particles decorated with a thin stabilizing shell of the excess component (PLL or CS), but no excessive PLL or CS amount. In the uncentrifuged case PEC dispersions contain both PEC particles with thin excessive PEL shells as well as the excess component. Thus, in coatings casted from uncentrifuged PEC dispersions either excessive PLL or CS is included in the polymer film and upon rinsing with water these excessive pure PEL compounds are readily dissolved. Presumably, this dissolution of pure PEL compounds may make the casted PEC particles also more prone to detachment.

### 3.2. Drug Loaded PEC

Since we have identified the PLL/CS-1.0 system as the most apt one with respect to redispersibility after centrifugation, we focused on this system for further studies concerning the loading with therapeutic drugs. In this subsection we evaluate the loading capacities of this PEC system with respect to the bisphosphonate risedronate (RIS) and the antibiotic rifampicin (RIF) based on charge considerations. Thereby the influence of the pH and the molar mixing ratio n^−^/n^+^ was studied. For the negatively charged RIS (pK_1_ = 1.6 ± 0.2, pK_2_ = 2.2 ± 0.2 pK_3_ = 5.9 ± 0.1, pK_4_ = 7.1 ± 0.1, pK_5_ = 11.7 ± 0.3) [23], the zwitterionic antibiotic rifampicin (pK_1_ = 1.7, pK_2_ = 7.9) [24] as well as PLL (pK_S_ = 10.5) [25] the individual pK_S_ values of its functional groups are known, which are important factors for electrostatic interactions. Furthermore, also the particle sizes of selected drug loaded PEC particles are presented in comparison to their unloaded analogues.

#### 3.2.1. Preparation Aspects

**Figure 6:**
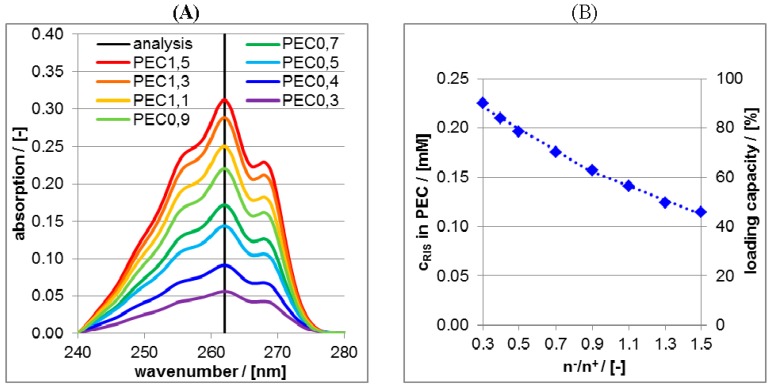
(**A**) Ultraviolet/visible (UV/VIS) spectra of supernatant after centrifugation of RIS-loaded PEC dispersions at pH = 10 for various molar mixing ratios n^−^/n^+^; (**B**) Plot of the concentrations (left ordinate axis) and of the loading capacity (right ordinate axis) of RIS in the PEC particles as a function of molar mixing ratio n^−^/n^+^.

In Figure 6A UV/VIS spectra of supernatants after the centrifugation of RIS/PLL/CS at pH = 10 are shown.

The intensities at the wavelength position of 262 nm were converted into concentrations and subtracted from the RIS bulk concentration of 0.25 mM, which is plotted against the molar mixing ratio n^−^/n^+^ of PEC in the Figure 6B. These ordinate values (left ordinate axis) are measures for the bound RIS amount into PEC particles and can further be converted to loading capacity values (right ordinate axis) by ratioing the bound RIS concentration and the initial RIS concentration. Obviously, the loading capacity increases approximately linearly with decreasing n^−^/n^+^ meaning, that the more positive the net charge of the PEC the higher is the RIS loading capacity. Vice versa the more negative the net charge the lower is the RIS loading capacity. This can be explained based on electrostatic considerations. According to the pKs values given above, RIS adopts two negative charges at pH = 10. Thus, dianionic RIS is electrostatically bound (“condensed”) at cationic PLL and therefore the loading capacity is highest for highest PLL content, which can be achieved for lowest n^−^/n^+^ values. In principle the binary PLL/RIS system (n^−^/n^+^ = 0) would lead to the highest RIS loading capacity. However, binary PLL/RIS systems have major disadvantages compared to ternary ones, which will be explained further below.

Obviously, RIS is already bound quantitatively by cationic PLL in the RIS/PLL mixture and cannot be replaced upon complexation by CS/RIS mixture, if the pH value is kept constant at pH = 10. This could be confirmed by PCD measurements on the PLL/RIS mixtures, which are presented in the Figure S1 of the Supplementary. There the charge factor F^+^ of PLL is plotted against the RIS concentration (c_RIS_) and it was observed, that F^+^ decreased linearly with increasing c_RIS_. Obviously, whenever RIS was complexed by PLL at pH = 10 the counterion condensation of RIS on PLL was so strong, that neither the titrator polyanion nor the CS could replace (evaporate) the anionic RIS. This is somewhat surprising, since PLL with pK_S_ = 10.5 [25] should be rather weakly charged at pH = 10 and not prone to bind RIS to such extent. As it is known PLL adopts at pH = 10 a rather stiff α-helical conformation [26]. There is theoretical evidence, that such charged cylinders are known to cause stronger counterion condensation [27], which might also prevail for PLL and the dianionic counterion RIS.

**Figure 7:**
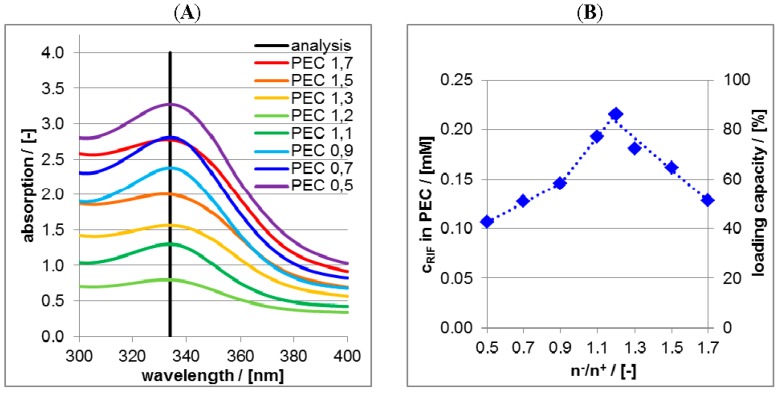
(**A**) UV/VIS spectra of supernatant after centrifugation of RIF-loaded PEC dispersions at pH = 2 for various molar mixing ratios n^−^/n^+^; (**B**) Plot of the concentrations (left ordinate axis) and of the loading capacity (right ordinate axis) of RIF in the PEC particles as a function of the molar mixing ratio n^−^/n^+^.

Analogously to RIS/PLL/CS in the Figure 6A UV/VIS spectra of supernatants after the centrifugation of RIF/PLL/CS dispersions at pH = 2 are shown in Figure 7A.

The intensities at the wavelength position of 340 nm were converted into RIF concentrations and further into the loading capacity and plotted against molar mixing ratios n^−^/n^+^ in the Figure 7B. Obviously the trend of Figure 7B concerning RIF is deviating from that of Figure 6B concerning RIS. For n^−^/n^+^ < 1.2 RIF loading increases with increasing CS concentration, a maximum of RIF loading is reached for n^−^/n^+^ = 1.2 and for n^−^/n^+^ > 1.2 RIF loading decreases. Again an explanation based on electrostatic considerations can be given. At pH = 2 zwitterionic RIF adopts a positive charge and with increasing n^−^/n^+^ (*i.e.*, anionic CS amount) RIF is increasingly loaded at PLL/CS, which is analogous to RIS loading at PLL/CS. However, for n^−^/n^+^ > 1.2 anionic CS is in excess and CS/RIF complexes can be formed. Unlike PLL/RIS complexes, these CS/RIF complexes are soluble and not phase separated after centrifugation, do not appear in the centrifuged pellet but in the supernatant and therefore do not contribute to the loading capacity. Hence, at n^−^/n^+^ = 1.2 the loading capacity of RIF in PLL/CS has a peak and diminishes at n^−^/n^+^ > 1.2.

The general trend for the binding of RIF at CS could be again confirmed by PCD measurements on the CS/RIF mixtures, which are presented in the Figure S2 of the Supplementary. There the charge factor F^−^ of CS is plotted against the RIF concentration (c_RIF_) and F^−^ decreased linearly with increasing c_RIF_. Obviously, whenever RIF was complexed by CS at pH = 2, the counterion condensation of RIF on CS was so strong, that neither the titrator polycation nor the PLL could replace (evaporate) the cationic RIF. This finding confirms the assumption, that for a large CS excess RIF/CS complexes are formed, although they could not be evidenced by UV/VIS spectroscopy (nonmonotonous behavior in Figure 7B), since they were soluble and could not be separated by centrifugation.

#### 3.2.2. Colloidal Properties

Analogously to the drug free PEC, DLS measurements were performed on the drug loaded PEC systems. The sizes of the drug loaded PEC particles are summarized in Table 3.

Generally, the particle sizes of centrifuged drug/PEC samples were larger compared to the drug free PEC systems (Table 2). In the case of RIS/PEC (1074 nm) they exceeded the 1 micron range, while in the case of RIF/PEC (592 nm) submicron particles were obtained. We explain the size increase of drug loaded compared to unloaded PEC particles by both an enhanced compensation of the excess charge at the PEC particles as well as a reduction of the Debye length in the PEC dispersion by the presence of low molecular charged drugs in the sense of counterions and coions, respectively. For RIS/PLL/CS-1.0 it is the positive net charge of the PEC 0.9, which is compensated by negatively charged RIS at pH = 10 and for RIF/PLL/CS-1.0 it is the negative net charge of PEC 1.2, which is compensated by positively charged RIF at pH = 2. These effects decrease the mutual repulsion of PEC particles and result in PEC particle aggregation.

**Figure 8:**
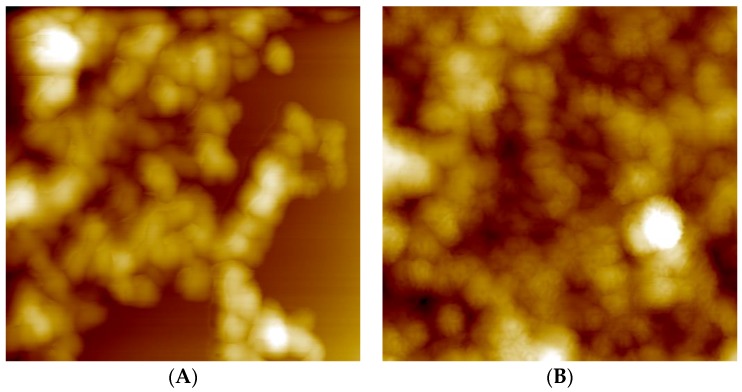
(**A**) 2 × 2 µm scanning force microscopy (SFM) image of PLL/CS-1.0 (PEC 0.9) after centrifugation step; (**B**) 2 × 2 µm SFM picture of RIS/PLL/CS-1.0 (RIS/PEC 0.9) after centrifugation step.

Furthermore, an explanation for the larger size of RIS/PEC compared to RIF/PEC particles could be the additional electrostatic crosslinking or bridging of PEC particles at their outermost PLL shell by RIS, which is a dianion at pH = 10, while RIF is monocation at pH = 2. Further investigations on particle size and shape were performed using scanning force microscopy (SFM). SFM images of centrifuged and redispersed unloaded (Figure 8A) and RIS-loaded (Figure 8B) PEC 0.9 particles are shown in Figure 8.

Unloaded PEC particles (Figure 8A) show spherical shape and diameters of around 150 nm. These diameters are significantly smaller than the hydrodynamic diameters determined by DLS (see Table 2). This deviation is due to water loss and shrinking of the PEC particles upon drying. Interestingly, SFM gives evidence, that the size and shape of RIS loaded PEC particles is similar to unloaded ones (Figure 8B), while DLS indicates significantly larger hydrodynamic diameters of RIS loaded PEC particles in comparison to drug‑free ones (Table 2 and Table 3). Obviously, RIS loaded PEC particles consist of micron sized aggregates (DLS), in which individual rather nano-sized particles can be identified (SFM). Those aggregates might be stabilized by crosslinks between RIS and the excess component PLL situated in the shell of the smaller PEC particles.

### 3.3. Drug Release from PEC Coatings at Model Substrate

Most importantly, the drug release properties of the drug loaded PEC coatings were studied at germanium model substrates. In this subsection, the influence of the pH and of ionic strength on the release of the two drugs RIS and RIF are treated. Herein we concentrate on the PEC 0.9 system for RIS release and the PEC 1.2 system for RIF release, since these n^−^/n^+^ settings were identified as most effective with respect to loading in the previous section. Drug release was determined by both ATR-FTIR spectroscopy, which is sensitive to the drug depletion in the PEC coating [13], and UV/VIS spectroscopy, which is sensitive to the drug enrichment in the release medium.

#### 3.3.1. Pure Drug Release

**Figure 9:**
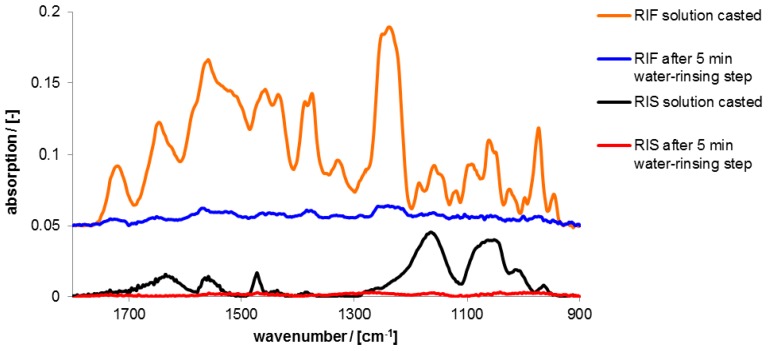
ATR-FTIR spectra of RIS and RIF films casted from 200 µL solutions (0.002 M) of the pure drug in the initial dry state and the dry state after 5 min rinsing in 2-(4-(2-Hydroxyethyl)-1-piperazinyl)-ethane sulfonic acid (HEPES) solution.

In the Figure 9 ATR-FTIR spectra of solution casted pure RIS and RIF coatings are shown in the initial dry state and the dry state after rinsing with HEPES buffer.

Prominent infrared (IR) peaks of these spectra together with those of the used polyelectrolytes are assigned in the Table S1 of the Supplementary. Evidently, films of both drugs RIS and RIF without PEC were immediately dissolved after contact to release medium without retention. Nearly 100% of the drugs were released within 5 min.

#### 3.3.2. Effect of pH on Drug Release

##### Risedronate/PEC

**Figure 10:**
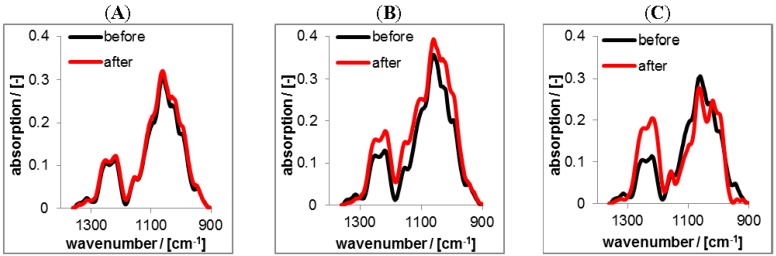
ATR-FTIR spectra of solution casted RIS loaded PEC 0.9 coatings prepared at pH = 10 (after centrifugation procedure) before (black) and after (red) rinsing (2 days) with tris(hydroxymethyl)-aminomethane (TRIS) at pH = 10 (**A**); HEPES at pH = 7 (**B**) and CIT at pH = 4 (**C**).

In Figure 10 typical ATR-FTIR spectra of solution casted RIS loaded PEC 0.9 particles of PLL/CS-1.0 for various release conditions (pH values) are shown in the range between 900 and 1400 cm^−1^. The obtained peaks are assigned in the Table S1 of the Supplementary.

**Figure 11:**
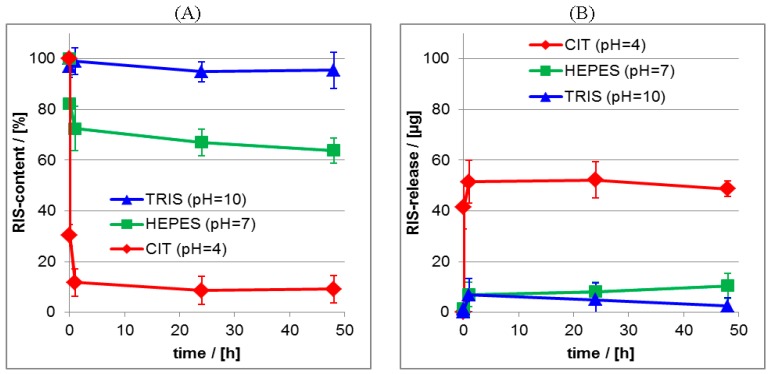
(**A**) Release kinetics from RIS/PEC films at aqueous release media for pH = 4, pH = 7 and pH = 10. (ATR-FTIR data); (**B**) Release kinetics from RIS/PEC films at aqueous release media for pH = 4, pH = 7 and pH = 10. (UV/VIS data).

RIS loaded PEC dispersions were prepared, centrifuged and redispersed at pH = 10 and solution casted at Ge IRE. Black spectra in Figure 10 represent the RIS loaded PEC film in the initial dry state and red spectra those PEC films in the dry state after rinsing (2 days) in various buffer solutions of citrate, HEPES or TRIS, respectively. Comparing the spectra of the initial dry state and that after rinsing, there are no changes visible for the rinse at pH = 10 (TRIS), which means qualitatively, that no or only low amount of RIS has been released. Spectral changes are only found for pH = 7 (HEPES) and pH = 4 (CIT). For pH = 4 an evident decrease of the composed band at around 1093 cm^−1^ is visible. Components of this band are originating from both the υ(O=P=O) vibration of the bisphosphonate group of RIS and the υ(C–O) vibration of saccharide moieties (ether linkages) of CS. Whereas, also an increase of the composed band at around 1250 cm^−1^ is visible, which originates solely from the υ(O=S=O) vibration and indicates a concentration increase of CS. Therefore, we conclude that for pH = 4 RIS is released and CS representative for the PEC phase is filling the space previously occupied by RIS. For pH = 7 the composed band at 1093 cm^−1^ does not show a clear trend, since the concentration increase of the PEC film (saccharide moieties) compensates the concentration loss of RIS (bisphosphonate groups). For the further quantification of drug release lineshape analysis was used, as it is described in the Experimental Section. The results are given in the Figure 11A.

Significantly, the release at pH = 4 resulted in the highest initial burst and lowest residual RIS content (≈10%), while under neutral and basic conditions lower initial bursts and higher residual contents were obtained (≈70%–90%). The same trend as for the ATR-FTIR data was obtained for the UV/VIS data given in the Figure 11B, where it is shown, that at pH = 4 nearly 60 μg of RIS were released, while at pH = 7 and pH = 10 around 10 μg of RIS were released after 24 h.

In conclusion, with decreasing pH value of the release medium an increasing RIS release was found. In other words, the greater the deviation between the pH value applied for preparation and the pH value applied for release, the stronger and faster was the RIS release. This trend was confirmed by UV/VIS measurements on the release media. We explain this effect considering electrostatic interactions between RIS and PLL. For the release at pH = 10 the amount of charges of both compounds does not change with respect to the initial state. Thus, there is no RIS release, because of the strong electrostatic interactions between RIS and PLL. Note, that even at pH = 10 PLL is still positively charged due to its pK_S_ value of 10.5 [25]. Upon changing the pH value in the release medium with respect to the pH value at preparation, also the amount of RIS charges decreases with decreasing pH value causing increasing RIS release. Obviously, it is the charge state of RIS and the related electrostatic interactions, which determine the binding to PLL and the release kinetics.

##### Rifampicin/PEC

**Figure 12:**
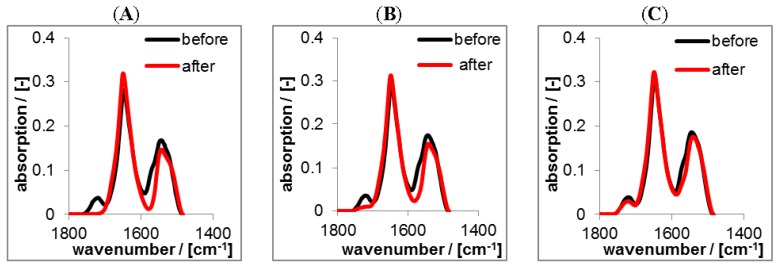
ATR-FTIR spectra of solution casted RIF-loaded PEC1.2 films prepared at pH = 2 before and after rinsing (2 days) with TRIS at pH = 10 (**A**); HEPES at pH = 7 (**B**) and CIT at pH = 4 (**C**) in the dry state, respectively.

Analogous release studies were performed on RIF/PEC 1.2 films. While RIS/PEC films were advantageously prepared at pH = 10 to get a high negative charge of RIS (see above), RIF/PEC films were prepared at pH = 2, since at this pH setting zwitterionic RIF acquires a positive charge. The ATR-FTIR spectra in the diagnostic range between 1800 and 1500 cm^−1^ are given in the Figure 12 featuring an isolated band at around 1725 cm^−1^, which can be assigned to the υ(C=O) vibration of either acetyl or lactone functional groups of RIF (Table S1).

Again, the black spectra represent the RIF/PEC films in the initial dry state at pH = 2. The red spectra in Figure 12 represent PEC films in the dry state after rinsing at pH = 4, pH = 7 and pH = 10. For the release at pH = 4 the spectra before and after rinse were nearly identical, only the band at 1725 cm^−1^ diagnostic for RIF decreased slightly after rinsing. In contrast for pH = 7 and pH = 10 the intensity of this band significantly decreased. This finding can be explained analogously to the RIS/PEC system, but in an opposite sense. Since RIF has zwitterionic properties and CS is a strong polyanion, RIF acquires considerable amount of positive charge, while CS is negatively charged at pH = 4, and thus electrostatic attraction should prevail. Increasing the pH value RIF gets less positively and more negatively charged, while the charge density of CS is invariant. Hence, at pH = 7 electrostatic attraction between RIF and CS decreases and at pH = 10 it is minimum and turns into electrostatic repulsion. In contrast to the FTIR spectra on the RIS/PEC system those of the RIF/PEC system do not show intensity variations of bands diagnostic for PLL or CS. Less loaded drug amounts might be the cause for the RIF/PEC system.

**Figure 13:**
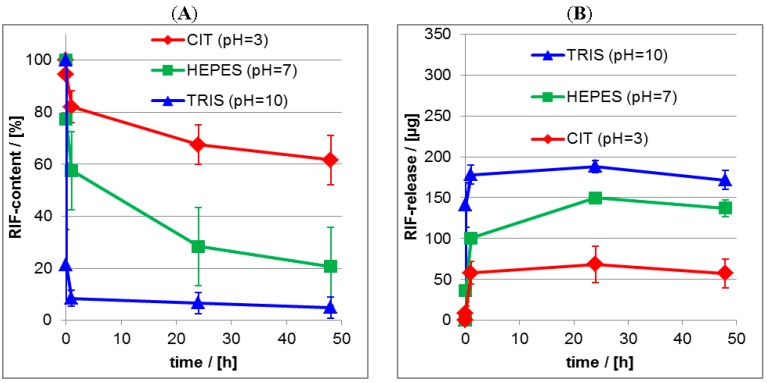
(**A**) Release kinetics from RIF/PEC films at aqueous release media for pH = 4, pH = 7 and pH = 10 as measured by ATR-FTIR spectroscopy; (**B**) Release kinetics from RIF/PEC films at aqueous release media for pH = 4, pH = 7 and pH = 10 as measured by UV/VIS spectroscopy.

For the quantitative determination of the released drug amount lineshape analysis (LSA) was applied as it is described in the Experimental Section 2.5.2, which results in the actual relative (percentage) drug content of the PEC layer. In the Figure 13A,B the release kinetics of RIS and RIF from PLL/CS PEC films is compared for the three pH values. Furthermore, we compare the ATR-FTIR measurements, which monitor the actual drug content (%) in the PEC layer (its loss) with the UV/VIS measurements, were the released drug (μg) in the release medium (its enrichment) was detected. The equilibrium between drug in the PEC film and drug in the release medium sets in rather fast. Generally, after 1 day the release is finished and therefore a nearly constant residual drug content in the PEC film is available. These trends were confirmed by UV/VIS measurements of the release media. Obviously, the release is strongly dependent on the pH value, as it was already found by the qualitative comparison of the FTIR spectra in Figure 10 and Figure 12. The quantitative analysis shows, that the residual drug content for the RIS/PEC system decreases from around 90% at pH = 10 to around 10% at pH = 4. For the RIF/PEC system the residual drug content decreases from around 70% at pH = 4 to around 10% at pH = 10. These values are summarized in the Table 4.

Since the pH value directly influences the charge state of the drug and the used polyelectrolytes we emphasize electrostatic interactions as the main driving force for the release of charged drugs from the PEC films used herein, which has been outlined above. For medical applications of drug delivery systems like the herein emphasized PEC systems, the release behavior at pH = 10 or pH = 4 is not that relevant for the clinical situation encountered in bone surgery. In this case, pH = 7 is most relevant. Concerning to this condition the residual content of around 65% RIS achieved herein, is a clear improvement and very promising. In former reports we obtained residual drug contents of only 30% for zoledronate (ZOL), which is like RIS a further widely used bisphosphonate [20].

The release studies on RIF from PLL/CS system at pH = 7 did not result in such a good retention compared to RIS. Residual contents of around 20% for RIF were obtained under this pH condition. This different behavior might be explained by the zwitterionic character of RIF allowing to reverse its net charge sign from positive at pH = 4 to negative at pH = 10, while the negative net charge of RIS can be only neutralized by decreasing the pH from 10 to 4, but not reversed. Hence, for the RIS/PEC system electrostatic attraction can be tuned gradually, while for the RIF/PEC system electrostatic attraction can be turned step-like into repulsion, upon varying the pH value, which seems to be responsible for the lower retention of RIF at pH = 7.

#### 3.3.3. Effect of Salt

**Figure 14:**
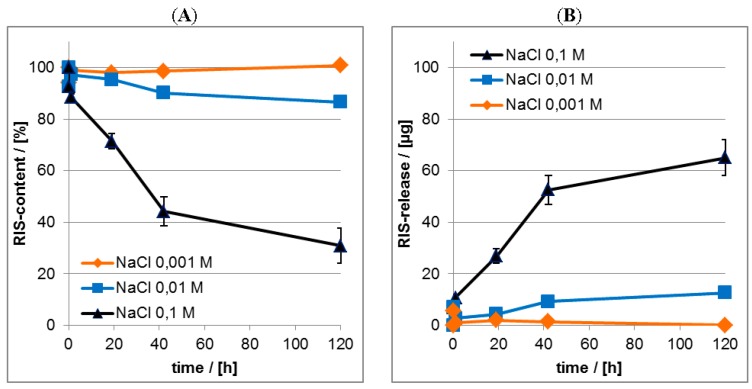
(**A**) Release kinetics from RIS/PEC in dependence of salt concentration—ATR-FTIR data; (**B**) Release kinetics from RIS/PEC in dependence of salt concentration—UV/VIS data.

In the Figure 14 results on the release kinetics of RIS from PEC coatings in dependence of NaCl concentration in the release medium determined by both ATR-FTIR (Figure 14A) and UV/VIS spectroscopy (Figure 14B) are shown. Herein we compare the RIS loss of the PEC films (ATR-FTIR) with the RIS enrichment in the release medium (UV/VIS). The UV/VIS data were shown as RIS masses (µg) in the release media.

Qualitatively, the release of RIS is enhanced by higher salt concentration in the release medium. This effect confirms our concept of electrostatically mediated interaction between PLL and RIS, since increasing ionic strength reduces the so called Debye length, which is around 1 nm for 0.1 M NaCl [28] meaning that electrostatic interaction does not prevail at drug/PEC distances exceeding 1 nm. The exact initial burst and residual content values in dependence of salt concentration are shown in Table 5.

### 3.4. Drug Release and Biocompatibility of PEC NP Coatings at Implant Material

In this final chapter, the RIS/PEC coatings characterized and validated at Ge model substrates were applied at Ti40Nb alloys, which is a relevant base material for the development of osteosynthetic plates due to its supreme mechanical properties [15,29].

#### 3.4.1. Drug Release

**Figure 15:**
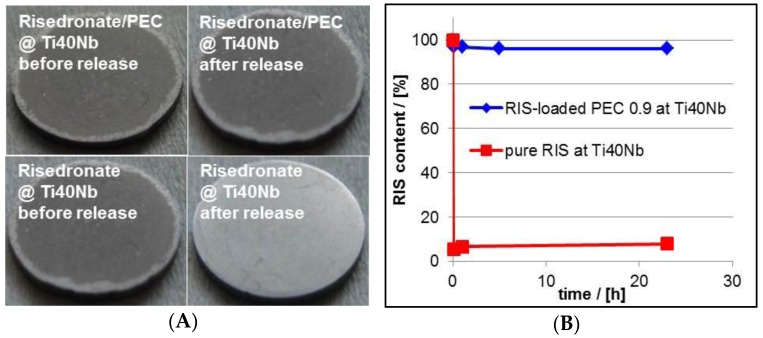
(**A**) Photographs of RIS/PEC- and RIS-modified Ti40Nb alloy plates before and after release experiment (PEC: PLL/CS-1.0). (**A**) Release kinetics of RIS from RIS/PEC modified Ti40Nb alloy plates measured by UV/VIS spectroscopy (**B**).

Based on our findings at the germanium model substrates we performed analogous measurements on drug release at relevant BSM. Herein, PEC and drug coatings were casted onto Ti40Nb alloy material. Photographs of typical RIS/PEC- and RIS-coated Ti40Nb alloy plates and the measured release kinetics at these implant materials are given in Figure 15.

As expected the total RIS amount from pure RIS film released in a very short time. Whereas, the RIS/PEC was adhesively stable and showed a very strong RIS retardation during the release experiment. Finally, the behavior of drug loaded PEC films casted onto implant materials was nearly the same compared to that at Ge model substrates, which can be found also in the Table 4. The slightly higher RIS retardation at Ti40Nb is explainable by the higher film thickness in comparison to that of the PEC film at Ge IRE, so that higher diffusion time within the coatings of Ti40Nb might play a role.

#### 3.4.2. Biocompatibility

Finally, the biocompatibility of the RIS/PEC coatings at the Ti40Nb alloys was studied. Human mesenchymal stem cells (hMSC), which are precursor cells of osteoblasts, were cultured onto bare Ti40Nb alloys and onto unloaded and RIS loaded PEC coatings of which and their morphology was investigated after 24 and 48 h of culturing time by digital photography. A set of digital photographs is presented in the Figure 15.

**Figure 16:**
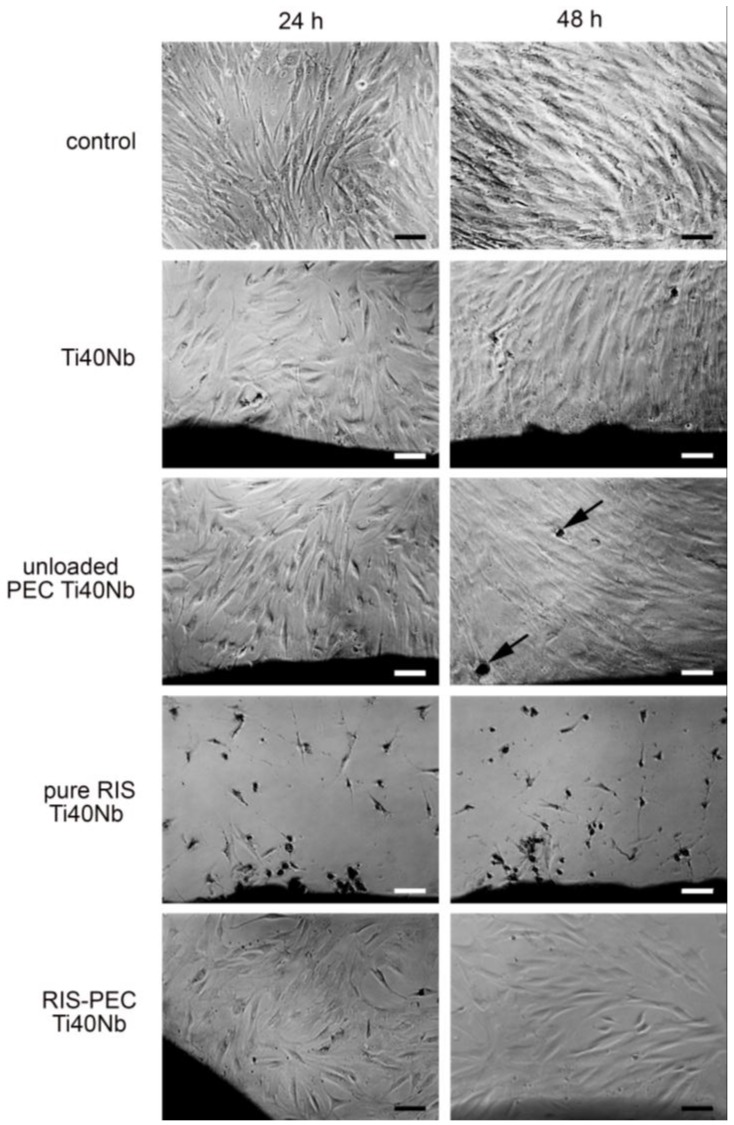
Biocompatibility of human mesenchymal stem cells (hMSC) on RIS loaded PEC at Ti40Nb after incubation of 24 and 48 h and its controls monitored by digital photography (scale bars: 100 μm).

Cells seeded on pure Ti40Nb alloys provided a lower cell number at 24 h as the controls growing on pure cell culture plastic. After 48 h hMSC on Ti40Nb showed a similar number and morphology like hMSC on pure plastic. Similar appearance was found for cells growing at the interface of unloaded PEC at Ti40Nb. At 48 h some of these cells already demonstrated signs of mineral production (Figure 15, arrow). On the other side cellular density of hMSC on the pure RIS film at Ti40Nb was decreased and the morphology revealed an unhealthy appearance. The hMSCs retracted their profiles and built up a spheroidal shape. hMSC on RIS-loaded PEC at Ti40Nb had a healthy appearance after 24 and 48 h of incubation. However at 48 h the cell number decreased which might be primarily resulted by the toxicity of RIS (Figure 16).

## 4. Conclusions

An improved interfacial drug delivery system (DDS) for osteotherapeutic drugs based on polyelectrolyte complex (PEC) coatings was elaborated. The cationic homopolypeptide poly(l-lysine) (PLL) was complexed by a mixture of two cellulose sulfates (CS) with low and high degree of substitution so that the CS and PLL solution have around equal molar charged units. As drugs the antibiotic rifampicin (RIF) and the bisphosphonate risedronate (RIS) were integrated. As an important advantage over previous ones this PEC system can be centrifuged, the supernatant discarded, the dense pellet phase (coacervate) separated, and again redispersed in fresh water phase. This behavior was shown to have three major advantages: (i) Access to the loading capacity of the drug, since the concentration of the free drug can be measured by spectroscopy; (ii) lower initial burst and higher residual amount of drug due to removal of unbound drug and (iii) complete adhesive stability due to the removal of PEL excess component. It was found that the pH value and ionic strength strongly affected drug content and release of RIS and RIF, so that we outline electrostatic interactions between drug and PEC as key interaction forces. Finally, studies at the clinically relevant implant material Ti40Nb revealed similar PEC adhesive and drug release properties compared to the model substrate germanium. Unloaded PEC coatings at Ti40Nb showed a similar number and morphology of above cultivated human mesenchymal stem cells (hMSC) compared to uncoated Ti40Nb and resulted in considerable production of bone mineral. RIS loaded PEC coatings showed similar effects after 24 h but resulted in reduced number and unhealthy appearance of hMSC after 48 h due to cell toxicity of RIS.

## Figures and Tables

**Table 1:** Charge factors and signs of 0.002 M polyelectrolyte (PEL) solutions.

PEL Sample	Factor F^+/−^	Charge Sign	Degree of Substitution (DS) (Supplier)
PLL	1.004 ± 0.025	(+)	1.0
CS-0.5	0.401 ± 0.016	(−)	0.5
CS-3.0	3.046 ± 0.030	(−)	3.0
CS-1.0 *	1.035 ± 0.020	(−)	1.0

* Mixture of CS-0.5/CS-3.0 ≈ 3.5:1 (volume).

**Table 2:** DLS results of drug free PEC particles, comparison of DLS parameters of PEC dispersions before and after centrifugation procedure.

Sample		Countrate (kHz)	Hydrodynamic Radius *R*_H_ (nm)	Polydispersity Index
PEC 0.9 PLL/CS-0.5	before	70 ± 3	131 ± 69	0.37 ± 0.03
	after	56 ± 11	235 ± 86	0.50 ± 0.02
PEC 1.1 PLL/CS-0.5	before	67 ± 10	211 ± 107	0.48 ± 0.01
	after	56 ± 3	229 ± 65	0.48 ± 0.03
PEC 0.9 PLL/CS-1.0	before	127 ± 14	98 ± 15	0.20 ± 0.02
	after	112 ± 12	121 ± 36	0.35 ± 0.10
PEC 1.1 PLL/CS-1.0	before	135 ± 18	133 ± 15	0.28 ± 0.08
	after	125 ± 18	157 ± 32	0.39 ± 0.10
PEC 0.9 PLL/CS-3.0	before	185 ± 14	100 ± 18	0.21 ± 0.01
	after	123 ± 9	155 ± 45	0.39 ± 0.02
PEC 1.1 PLL/CS-3.0	before	202 ± 24	109 ± 25	0.26 ± 0.05
	after	135 ± 3	236 ± 64	0.49 ± 0.24

**Table 3:** DLS parameters countrate and hydrodynamic radius of drug loaded PLL/CS particles.

Sample	Countrate (kHz)	Hydrodynamic Radius *R*_H_ (nm)	Polydispersity Index
RIS/[PLL/CS-1.0]-0.9	54 ± 6	1074 ± 420	0.39 ± 0.06
RIF/[PLL/CS-1.0]-1.2	43 ± 2	592 ± 41	0.32 ± 0.03

**Table 4:** Comparison of initial burst (IB) and residual content (RC) for drug/PEC in dependence of pH value of release media.

pH of Release Medium	RIS/REC 0.9		RIF/PEC 1.2	
	IB (%)	RC (%)	IB (%)	RC (%)
pH = 4	70 ± 4	9 ± 5	5 ± 4	62 ± 9
pH = 7	18 ± 11	64 ± 5	23 ± 17	21 ± 15
pH = 10	3 ± 4	96 ± 7	79 ± 13	5 ± 4

**Table 5:** Comparison of initial burst (IB) and residual content (RC) for RIS/PEC in dependence of salt concentration of release media.

Salt Concentration of Release Media	RIS/PEC 0.9	
	IB (%)	RC (%)
0.001 M	6 ± 1	100 ± 1
0.01 M	7 ± 1	87 ± 2
0.1 M	7 ± 1	31 ± 7

## Supplementary Materials

The following are available online at http://www.mdpi.com/2079-4991/6/3/53/s1.
