# Supplementary material for: Polyelectrolyte Complex Based Interfacial Drug Delivery System with Controlled Loading and Improved Release Performance for Bone Therapeutics
Source: Nanomaterials (Basel). 2016 Mar 22;6(3):53. doi: 10.3390/nano6030053 (PMC5302517; doi:10.3390/nano6030053)
Supplement: Supplementary file 1 [file nanomaterials-06-00053-s001.pdf]

Supplementary Materials: Polyelectrolyte Complex Based Interfacial Drug Delivery System with Controlled Loading and Improved Release Performance for Bone Therapeutics

David Vehlow, Romy Schmidt, Annett Gebert, Maximilian Siebert, Katrin Susanne Lips and Martin Müller

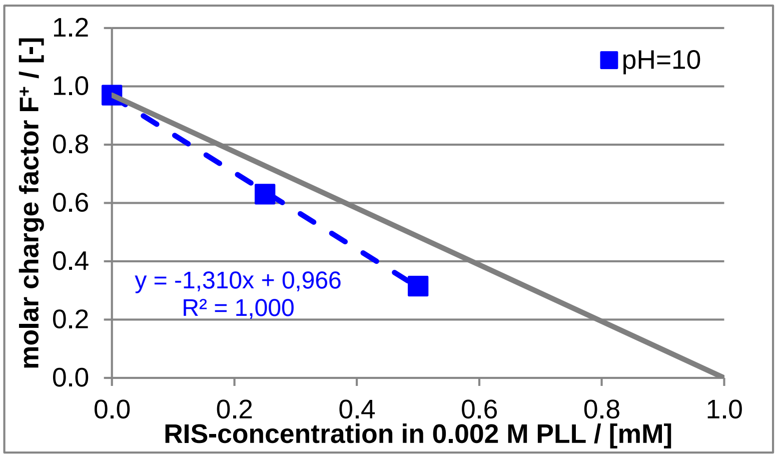

Figure S1. Molar charge factor of PLL at pH = 10 in dependence of RIS concentration in solution.

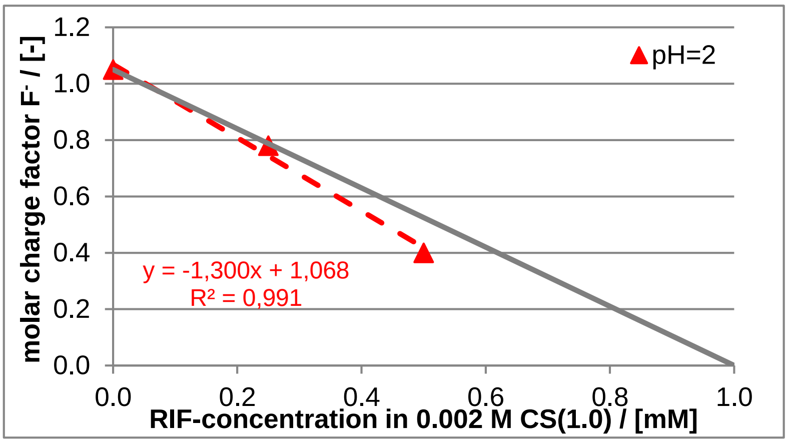

Figure S2. Molar charge units of CS-1.0 at pH = 2 in dependence of RIF concentration in solution.

Table S1. Assignments of diagnostic peaks in Fourier transform infrared (FTIR) spectra of drug loaded polyelectrolyte complex (PEC) coatings. IR: Infrared; RIF: Rifampicin; PLL: Poly(l-lysine); CS: Cellulose sulfates; RIS: Risedronate.

Peak (cm^{-1})	IR Vibration	Component
1725	ν(C=O)	RIF
1650	Amide I	PLL
1220	ν(O=S=O)	CS
1093	ν(O=P=O)	RIS

© 2016 by the authors; licensee MDPI, Basel, Switzerland. This article is an open access article distributed under the terms and conditions of the Creative Commons by Attribution (CC-BY) license (http://creativecommons.org/licenses/by/4.0/).
